# Changes in soil organic carbon and nitrogen stocks in organic farming practice and abandoned tea plantation

**DOI:** 10.1186/s40529-023-00401-z

**Published:** 2023-09-28

**Authors:** Kai-Wei Juang, Chiou-Pin Chen

**Affiliations:** 1 Department of Agronomy, National Chiayi University, No. 300, Syuefu Rd., 600355 Chiayi City, Taiwan; 2 The Experimental Forest, College of Bioresources and Agriculture, National Taiwan University, No. 12, Sec. 1, Qianshan Rd., 55750 Zhshan Township, Nantou County, Taiwan

**Keywords:** SOC stock, N stock, Secondary forest, Organic farming, Tea plantation

## Abstract

**Background:**

The restoration of conventional tea plantations and the adoption of organic farming practices could impact soil organic carbon (SOC) and nitrogen (N) stocks. This study investigated the soil properties, SOC and N contents and stocks, and their vertical distributions of a secondary forest restored from an abandoned conventional tea plantation and a converted organic tea plantation. An adjacent conventional tea plantation employing similar intermediate farming served as a comparison.

**Results:**

Within a 50-cm depth, the secondary forest exhibited a higher SOC stock of 115.53 ± 7.23 Mg C ha^− 1^ compared to 92.1 ± 8.54 Mg C ha^− 1^ for the conventional tea plantation. No significant differences in N stocks were seen between the two land uses. Significantly high SOC and N contents and stocks were found in the 0–10 cm layer of the secondary forest compared to the conventional tea plantation. No significant disparities in SOC and N stocks were found between the conventional and organic tea plantations within the 50 cm depth (92.1 ± 8.54 Mg C ha^− 1^ and 10.06 ± 1.01 Mg N ha^− 1^ vs. 97.47 ± 1.53 Mg C ha^− 1^ and 9.70 ± 0.10 Mg N ha^− 1^). However, higher levels of SOC and N contents and stocks were observed at a depth of 10 cm in the conventional tea plantation and below 10 cm in the organic tea plantation.

**Conclusions:**

The C and N inputs derived from high litter production at the top soil strongly contributed to higher SOC and N contents and stocks in the secondary forest. The application of soybean amendments in the conventional tea plantation and the longer tea plantation age of the organic tea plantation influenced their distribution of SOC and N contents and stocks, respectively. Reverting a conventional tea plantation into a secondary forest contributed to C recovery and reaccumulation. The conventional tea plantation, employing similar intermediate farming practices, increased SOC and N contents and stocks in the surface soil compared to the organic tea plantation. However, adopting organic farming did not significantly increase SOC stocks compared to the conventional tea plantation.

**Supplementary Information:**

The online version contains supplementary material available at 10.1186/s40529-023-00401-z.

## Background

Soil organic carbon (SOC) accumulation has an important effect on carbon (C) stocks and nutrient cycling in forest ecosystems as well as on global climate change and terrestrial C balance. C can be fixed in the form of biomass and soil C; over two-thirds of C is sequestered in soil and associated peat deposits in forest systems (Dixon et al. 1994). However, land-use changes can lead to a reduction in SOC stocks owing to various factors such as soil erosion, vegetation conversion, and forest conversion (Chen et al. 2004, 2016; Don et al. 2011; Fu et al. 2010; Schulp et al. 2008; Smith et al. 2002). Such land-use changes can negatively impact SOC accumulation and disrupt the natural C cycle within ecosystems.

Previous studies have shown that the conversion of natural forests to artificial forests resulted in a 13% decline in SOC stocks, while the transformation of forests into cultivated lands led to a more significant reduction of approximately 25–42% (Don et al. 2011; Guo and Gifford 2002; Murty et al. 2002). In addition to deforestation, farming activities such as cultivation, harvesting, weeding, and intensive tillage practices contribute to the decline in SOC and N stocks in cultivated land by accelerating the decomposition of soil organic matter (SOM) and reducing C levels (Chen et al. 2016; Guan et al. 2015).

Reforestation efforts can reverse the trend of C loss by promoting C recovery in the soil. Converting agricultural lands back to natural or perennial vegetation can reaccumulate C in the soil (DeGryze et al. 2004; Post and Kwon 2000), rebuilding SOC and N stocks. Additionally, external C inputs (e.g., soybean amendments, organic fertilizers, animal manures, green manures, and crop residues) and conservation management activities can also aid in increasing SOC and N stocks and alleviate their losses in cultivated land (Di et al. 2020; Gai et al. 2018; Kautsar et al. 2020; Khan et al. 2019; Shukla and Lal 2005; Su et al. 2022).

Tea (*Camellia sinensis* L.) is the second-most widely consumed beverage globally, after water (Chang 2015; Hicks 2001). It is primarily cultivated in mountainous regions with specific agroclimatic requirements, including a hot and moist climate, temperatures between 10 and 30 °C, and annual precipitation exceeding 1,250 mm (Chang 2015). Tea plantations are typically established on highly weathered soil with pH levels of 4.5–5.5 (Chang 2015; Zoysa et al. 1999). Global tea production exceeds 5,063,900 tons per year, with major tea-producing countries including China, India, Sri Lanka, Vietnam, Indonesia, Bangladesh, Kenya, and Turkey (Chang 2015). However, the pursuit of economic gains from tea cultivation often leads to the conversion of natural forests into tea plantations, resulting in ecological changes and the loss of valuable ecosystem services.

Nantou County in Central Taiwan has a warm, humid climate with abundant rainfall. It features extensive tea plantations covering approximately 6,552 hectares (53% of the plantation area) and contributing to 64% of the total tea production, which amounts to 7,372 tons annually. However, the aging population has resulted in a labor shortage for cultivation. Additionally, with the increasing emphasis on soil and water conservation, tea plantations are increasingly being reverted back to forests. Moreover, owing to the impacts of conventional farming and the goals of sustainable agriculture, the Taiwanese government has promoted organic farming since the 1990s, leading to the growing adoption of organic practices in the main tea-producing region. Therefore, in Central Taiwan, reverting abandoned tea plantations into natural forests and the adoption of organic farming practices in conventional tea plantations play significant roles in increasing soil C storage.

Despite the importance of reverting tea plantations to forests and implementing organic farming for C sequestration are crucial, limited quantitative research has focused on their specific effects on SOC stocks. Only a few studies have specifically examined and evaluated SOC and N stock within the context of tea plantations. Some studies have explored the impact of converting forests into tea plantations (e.g., Chen et al. 2016; Chiti et al. 2018; Sohng et al. 2017; Yüksek and Yüksek 2021; Yulnafatmawita et al. 2020). The reversion of tea plantations to forests or the implementation of different management practices has rarely been investigated in literature. Therefore, further research is required to understand the changes in SOC and N stocks resulting from these reversions and to assess their potential for C sequestration. The main objective of this study was to investigate and compare SOC and N stocks, along with their vertical distributions, in soil profiles of a broadleaved secondary forest restored from an abandoned conventional tea plantation and a converted organic tea plantation. Additionally, we selected an adjacent conventional tea plantation as a reference for comparison.

## Methods

### Study site description

**Fig. 1:**
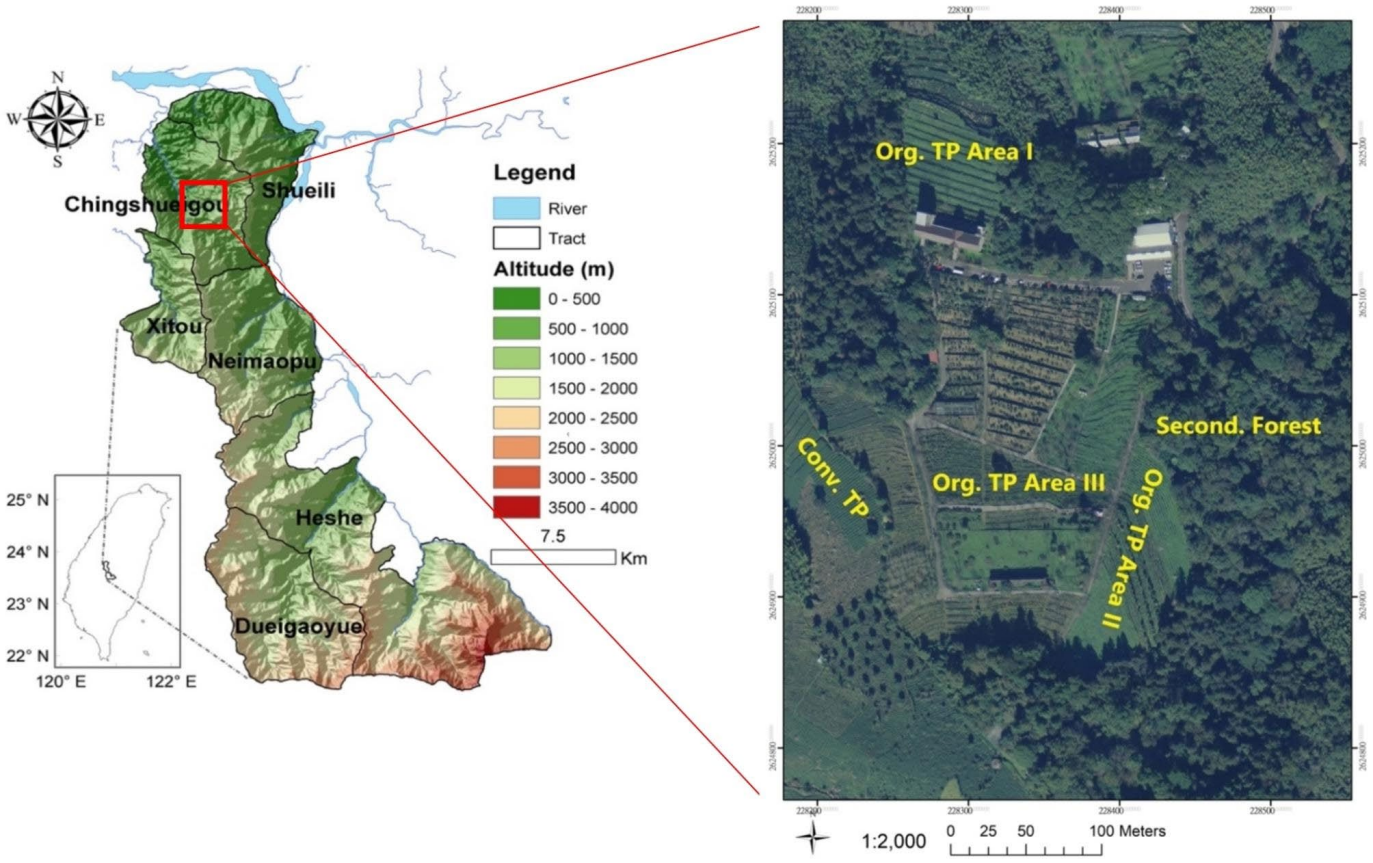
Study area in Central Taiwan. The geographic positions of the secondary forest (Second. Forest), the organic tea plantation (Org. TP Area I, II, III), and the adjacent private conventional tea plantation (Conv. TP) in Lugu Township, Nantou County, Taiwan

The study was conducted in March 2021 in Lugu Township, Nantou County, Central Taiwan (latitude 23°43′44″N; longitude 120°46′51″E), with an elevation of 800–900 m a.s.l. (Fig. 1). The region experiences an annual precipitation of 2,250 mm and has a mean monthly temperature of 18.5 °C. The parent materials in this area include sandstone, shale, and mudstone, and the soil is classified as an Inceptisol based on the USDA Soil Taxonomy (Soil Survey Staff 2014). Lugu Township is characterized by extensive tea plantations, with Oolong tea being the predominant cash crop known for its high quality and flavor. Many local tea plantations were established by converting natural broadleaved forests during the 1960s.

The study sites comprised an adjacent secondary forest, an organic tea plantation, and a private conventional tea plantation. The secondary forest and organic tea plantation are located in the Chingshueigou tract of The Experimental Forest, College of Bioresources and Agriculture, National Taiwan University (The Experimental Forest, NTU). In 1956, approximately 13 hectares of natural broadleaved forest was converted into conventional tea plantations. In 1972, a section of the tea plantation was abandoned, and over the course of 50 years, it underwent a natural succession process, leading to the development of the secondary forest. The secondary forest primarily consists of species such as Taiwan Acacia (*Acacia confusa*), Chinese guger tree (*Schima superba*), Taiwan paulownia (*Paulownia kawakamii*), tung tree (*Vernicia fordii*), turn-in-the-wind (*Mallotus paniculatus*), and Schefflera tree (*Schefflera octophylla*). The remaining conventional tea plantation continued to use conventional farming practices until switching to organic farming in 2014. Since the conversion, the tea plantation has received approximately 4,400 kg ha^− 1^yr^− 1^ of certified organic fertilizer (with a composition of N 4.9%, P_2_O_5_ 2.1%, K_2_O 1.9%, and OM 65%) for nutrient supplementation. The use of soybean meal fertilizer has been limited due to difficulties in obtaining non-genetically modified organism (GMO) certification. In conventional farming, chemical fertilizers and pesticides are extensively used to achieve higher yields per hectare (Azarbad 2022). However, unlike in traditional farming, farmers in Lugu Township commonly use a significant amount of soybean meal for tea plantation management due to the high N demand of tea plants, alongside chemical fertilizers and pesticides. This practice resembles intermediate farming practices and had been used in the organic tea plantation before its conversion in 2014.

A private conventional tea plantation adjacent to the secondary forest and organic tea plantation in the Chingshueigou tract of The Experimental Forest, NTU, was selected as the comparison site for this study. As of 2021, the conventional tea plantation has been managed using farming methods that are similar to intermediate farming practices as mentioned previously for nine years. Although the conventional tea plantation in this study falls under the category of conventional farming, the management of tea plantations differs from typical conventional farming owing to the specific practices employed by the local tea plantation farmers. The annual amendments in this conventional tea plantation include 4,500 kg ha^− 1^ of nitrophosphate organic compound fertilizer (N 20%, P_2_O_5_ 5%, K_2_O 10%, S 4.5%, CaO 5%, OM 60%), 2,400 kg ha^− 1^ of soybean meal fertilizer, and 560 kg ha^− 1^ of soybean flour. Additionally, periodic applications of pesticides and fungicides are carried out in this plantation.

The tea plant (*Camellia sinensis* L.) cultivated in both the organic tea plantation and the adjacent private conventional tea plantation is the Jhin-Hsuan (TTES no. 12) variety, which is highly popular and widely grown in Taiwan. Tea leaves are harvested 4–5 times a year, specifically during the spring, summer, autumn, winter, and late winter seasons, at both tea plantations.

### Soil sampling

In March 2021, soil sampling was carried out at each study site. A subplot measuring 20 × 25 m² was established in the adjacent secondary forest, organic tea plantation (Areas I, II, and III), and conventional tea plantation (Fig. 1). One subplot was designated within the secondary forest and conventional tea plantation, respectively. However, within the organic tea plantation, each of the three areas was assigned a subplot to minimize variations resulting from different regions. Within each subplot, three soil profiles were taken, resulting in a total of 15 soil profiles (3 profiles × 5 sites). The slopes of the selected profiles were less than 10° to reduce topographical effects. Before soil sampling, litterfall and weeds on the soil surface were cleared. Soil profiles were excavated to depths of 50–60 cm, considering the typically shallow soil development in this mountainous region.

To determination of bulk density (BD), three mineral soil samples were collected using volumetric cores at each depth of the soil profile before soil sampling. Soil samples were then collected at three depth intervals: 0–10 cm, 10–30 cm, and 30–50 cm. These collected soil samples were air-dried, ground, sieved to 2 mm, and stored for subsequent analyses. To estimate the percentage of rock fragment particles (stone%), another 1-kg soil sample was collected from each layer of the profiles. These samples were transported to the laboratory, air-dried, ground, and sieved through a 2-mm sieve. Finally, the separated soil and rock fragment particles were weighed to calculate their proportions for the calculation of SOC and N stock.

### Soil property analyses

The collected volumetric core soil samples were dried at 105 °C to a constant weight. BD was calculated by dividing the mass of oven-dried soil by the core volume (Blake and Hartge 1986). Soil texture and particle size distribution were determined using the pipette method (Gee and Bauder 1986). Soil pH was measured using a soil-to-water ratio of 1:1 with a glass electrode (McLean 1982). The air-dried soil was pulverized with a ball mill (Oscillating Mill MM400; Retsch, Newton, PA, USA). The ground soil samples were sieved to obtain a homogeneous particle size (< 100 mesh). Before analysis, the soil samples were further oven-dried at 105 °C overnight to eliminate any residual moisture. Subsequently, 30-mg soil samples were carefully weighed into foil capsules, and SOC and TN were determined using an elemental (CHN) analyzer following dry combustion (Perkin Elmer 2400 CHN; Perkin Elmer, Norwalk, CT, USA) (Chen et al. 2016). Each experiment was replicated three times, and the results for the organic tea plantation were reported as the mean of the data from the three areas.

### SOC and N Stock Estimation

The SOC and N stocks at a given soil depth were calculated using

1 $$Td = \sum\nolimits_{i = 1}^k {\rho iPiDi\left( {1 - Si} \right)}$$

where *Td* is the SOC or N stock over depth *d* of a layer (in Mg m^− 2^), *ρi* is the BD of layer *i* (in Mg m^− 3^), *Pi* is the SOC or TN content of layer *i* (in mg C g^− 1^ of soil), *Di* is the thickness of layer *i* (in m), and *Si* is the percentage of rock fragment particles > 2 mm in layer *i* (%) (Batjes 1996). The SOC and N stocks were summed from the measurements at soil depths of 0–10 cm, 10–30 cm, and 30–50 cm.

### Statistical analyses

The data for soil SOC, TN, C/N ratio, and SOC and N stocks in different layers were analyzed using one-way analysis of variance. Tukey’s post-hoc analysis was used to determine the difference between treatments, and p < 0.05 was considered statistically significant. All statistical analyses were performed using R software.

## Results

### Soil Properties

The findings of the soil property analyses are summarized in Table 1. Across all study sites, the soils were predominantly composed of sand and silt, with loam and clay loam textures observed in the surface soils and subsoils, respectively. As the soil depth increased, the proportion of sand decreased and that of clay increased. The BD also increased with depth across sites. The lowest BD (0.37 ± 0.02 Mg m^− 3^) was observed in the 0–10 cm layer of the secondary forest, and the highest BD (1.25 ± 0.20 Mg m^− 3^) was found in the 30–50 cm layer of the conventional tea plantation. In general, the BD was higher in the tea plantations than in the secondary forest, particularly in the upper 10-cm layer. Additionally, both tea plantations showed similar BD values across layers. The soil pH ranged from 3.1 ± 0.1 to 3.9 ± 0.0 across depths and sites. Similar to the BD, the soil pH increased with increasing soil depth at all study sites, showing an opposite trend to the SOC content (Table 2). The significant difference in soil pH among different land-use types was observed in the 0–10 cm soil layer, and the conventional tea plantation had the lowest soil pH.

**Table 1:** General soil properties at different depths in the secondary forest, the organic tea plantation, and the convnetional tea plantation

Study Site	Depth (cm)	Particle Distribution (%)			Texture	BD (Mg m^− 3^)	pH
		Sand	Silt	Clay			
Second. Forest	0–10	49.6	35.0	15.4	L	0.37 (0.02)	3.5 (0.1)
	10–30	36.3	34.8	29.0	L	0.87 (0.04)	3.7 (0.1)
	30–50	33.3	35.4	31.3	CL	1.07 (0.03)	3.9 (0.1)
Org. TP	0–10	46.3	34.9	20.7	L	0.82 (0.11)	3.6 (0.2)
	10–30	36.8	34.2	29.6	CL	1.00 (0.00)	3.7 (0.0)
	30–50	30.7	33.7	35.6	CL	1.15 (0.10)	3.9 (0.0)
Conv. TP	0–10	39.0	43.3	17.7	L	0.79 (0.07)	3.1 (0.1)
	10–30	31.8	34.9	33.3	CL	1.10 (0.10)	3.5 (0.3)
	30–50	22.9	34.8	42.3	CL	1.25 (0.20)	3.8 (0.3)

Second. Forest: Secondary Forest; Org. TP: Organic Tea Plantation; Conv. TP: Conventional Tea Plantation; L: Loam; CL: Clay Loam

### Vertical distribution of SOC, TN, and C/N ratio

The SOC and TN contents decreased with increasing soil depth (0–50 cm) at all sites (Table 2). The SOC contents ranged from 7.69 ± 0.59 to 160.95 ± 6.71 g kg^− 1^ and TN contents, from 1.01 ± 0.08 to 12.08 ± 0.28 g kg^− 1^, with the highest content seen in the 0–10 cm layer of the secondary forest and the lowest content seen in the 30–50 cm layer of the conventional tea plantation. Specifically, in the 0–10 cm layer, the SOC and TN contents in the secondary forest (160.95 ± 6.71 g kg^− 1^ and 12.08 ± 0.28 g kg^− 1^, respectively) were significantly higher than those in the conventional tea plantation (47.30 ± 4.95 g kg^− 1^ and 4.71 ± 0.58 g kg^− 1^, respectively). The conventional tea plantation had higher SOC and TN contents compared to those of the organic tea plantation (34.30 ± 1.91 g kg^− 1^ and 3.35 ± 0.27 g kg^− 1^, respectively). Although the SOC and N contents in the organic tea plantation were slightly higher than those in the conventional tea plantation at depths below 10 cm, no statistically significant difference was seen between the two tea plantations in the 10–30 cm layer.

The secondary forest had the highest C/N ratio (13.30 ± 0.85 in the 0–10 cm layer), and the conventional tea plantation had the lowest C/N ratio (7.61 ± 0.28 in the 30–50 cm layer) (Table 2). Excluding the extreme values in the 0–10 cm soil of the secondary forest and 30–50 cm soil of the conventional tea plantation, the C/N ratios across depths and sites ranged from 9.34 to 10.43. When comparing the C/N ratios at different depths across land-use types, the 0–10 cm soil layer showed significantly higher C/N ratios in the secondary forest compared to the conventional tea plantation, with no significant difference found between the organic tea plantation and the conventional tea plantation.

**Fig. 2:**
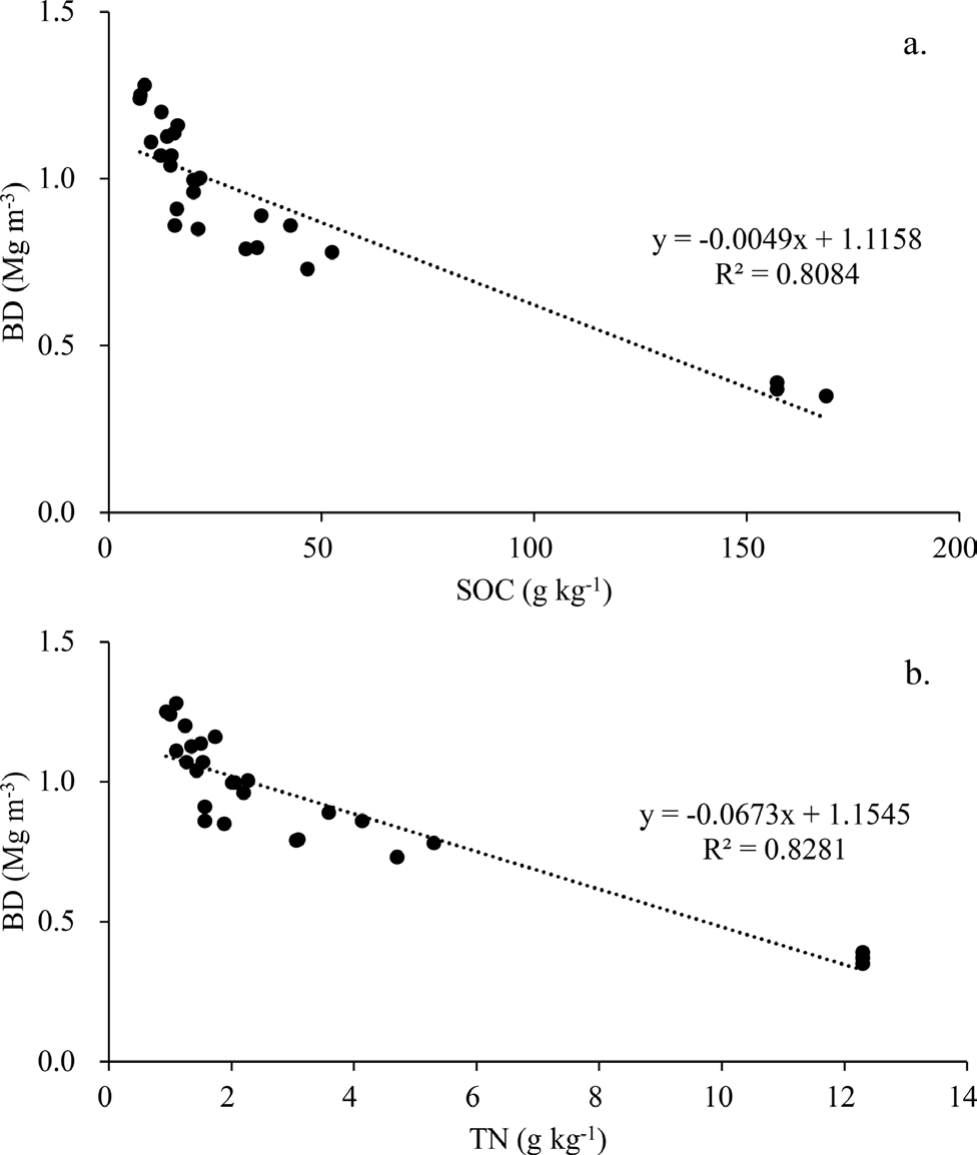
Relationship between **(a)** BD and SOC and **(b)** BD and TN across soil layers and land-use types

However, moving deeper into the 10–30 cm and 30–50 cm layers, the distinctions in C/N ratios among different land-use types became less pronounced, except for a low value observed in the 30–50 cm layer of the conventional tea plantation. Strong negative correlations were found between BD and the SOC (R^2^ = 0.8084) and TN (R^2^ = 0.8281) contents (Fig. 2a and b, respectively).

**Table 2:** SOC, TN, and C/N ratios at different depths in the secondary forest, the organic tea plantation, and the organic tea plantation

Depth (cm)	Study Site	SOC (g kg^− 1^)	TN (g kg^− 1^)	C/N
0–10 cm	Second	160.95	12.08	13.30
	Forest	(6.71)^a^	(0.28)^a^	0.85)^a^
	Org. TP	34.30 (1.91)^c^	3.35 (0.27)^c^	10.26 (0.27)^b^
	Conv. TP	47.30 (4.95)^b^	4.71 (0.58)^b^	10.06 (0.24)^b^
10–30 cm	Second	17.50	1.67	10.43
	Forest	(3.04)^a^	(0.18)^a^	(0.67)^a^
	Org. TP	20.62 (0.76)^a^	2.10 (0.12)^a^	9.81 (0.34)^ab^
	Conv. TP	16.96 (2.69)^a^	1.82 (0.34)^a^	9.34 (0.26)^b^
30–50 cm	Second	12.20	1.27	9.58
	Forest	(2.32)^a^	(0.17)^ab^	(0.58)^a^
	Org. TP	13.80 (1.47)^a^	1.37 (0.14)^a^	10.06 (0.13)^a^
	Conv. TP	7.69 (0.59)^b^	1.01 (0.08)^b^	7.61 (0.28)^b^

Second. Forest: Secondary Forest; Org. TP: Organic Tea Plantation; Conv. TP: Conventional Tea Plantation

Different letters in the same soil layer indicate significant differences among different land-use types at P < 0.05 (n = 3)

### Vertical distribution of SOC and N stocks

Within a 50-cm depth in the secondary forest, organic tea plantation, and conventional tea plantation, the SOC stocks were respectively 115.53 ± 7.23 Mg C ha^− 1^, 97.47 ± 1.53 Mg C ha^− 1^, and 92.1 ± 8.54 Mg C ha^− 1^, and the N stocks were respectively 10.12 ± 0.56 Mg N ha^− 1^, 9.70 ± 0.10 Mg N ha^− 1^, and 10.06 ± 1.01 Mg N ha^− 1^ (see Table 3). The SOC stock in the secondary forest was significantly higher than the conventional tea plantation, whereas the N stocks did not show significant differences between the two land use types. The SOC stock in the organic tea plantation was not significantly different from that in the conventional tea plantation; similarly, the N stocks did not show significant differences.

In the 0–10 cm layer, the secondary forest had higher SOC and N stocks (59.42 ± 1.61 Mg C ha^− 1^ and 4.55 ± 0.25 Mg N ha^− 1^, respectively) compared to those in the conventional tea plantations (37.16 ± 3.49 Mg C ha^− 1^ and 3.70 ± 0.38 Mg N ha^− 1^, respectively). However, in the 10–30 cm layer, the secondary forest had relatively lower SOC and N stocks (30.17 ± 4.96 Mg C ha^− 1^ and 2.88 ± 0.29 Mg N ha^− 1^, respectively). Deeper, in the 30–50 cm layer, the SOC stock was slightly higher than that in the conventional tea plantation, whereas the N stocks did not show significant differences.

Significant differences in SOC and N stocks between the two tea plantations were observed in the top layer. In the 0–10 cm layer, the conventional tea plantation had higher SOC and N stocks (37.16 ± 3.49 Mg C ha^− 1^ and 3.70 ± 0.38 Mg N ha^− 1^, respectively) compared to those in the organic tea plantation (27.36 ± 3.08 Mg C ha^− 1^ and 2.57 ± 0.45 Mg N ha^− 1^, respectively). In contrast, the organic tea plantation had relatively higher SOC stocks than the conventional tea plantation in the 10–30 cm and 30–50 cm layer (40.63 ± 1.43 Mg C ha^− 1^ and 29.48 ± 3.18 Mg C ha^− 1^, respectively). However, the two tea plantations did not show significant differences in N stock.

**Table 3:** SOC and N stocks (Mg ha^–1^) at different soil depths in the secondary forest, the organic tea plantation, and the conventional tea plantation

Depth (cm)	SOC Stock (Mg C ha^–1^)		
	Second Forest	Org. TP	Conv. TP
0–10	59.42(1.61)^a^	27.36 (3.08)^c^	37.16 (3.49)^b^
10–30	30.17 (4.96)^b^	40.63 (1.43)^a^	35.60 (3.65)^ab^
30–50	25.94 (4.13)^a^	29.48 (3.18)^a^	19.35 (1.85)^b^
**Total**	**115.53 (7.23)** ^**a**^	**97.47 (1.53)** ^**b**^	**92.1 (8.54)** ^**b**^
Depth (cm)	**N Stock (Mg N ha**^**–1**^)		
	Second Forest	Org. TP	Conv. TP
0–10	4.55 (0.25)^a^	2.57 (0.45)^c^	3.70 (0.38)^b^
10–30	2.88 (0.29)^b^	4.16 (0.27)^a^	3.83 (0.49)^a^
30–50	2.70 (0.27)^a^	2.97 (0.29)^a^	2.54 (0.25)^a^
**Total**	**10.12 (0.56)** ^**a**^	**9.70 (0.19)** ^**a**^	**10.06 (1.01)** ^**a**^

Second. Forest: Secondary Forest; Org. TP: Organic Tea Plantation; Conv. TP: Conventional Tea Plantation

Different letters in the same soil layer indicate significant differences among different land-use types at P < 0.05 (n = 3)

**Fig. 3:**
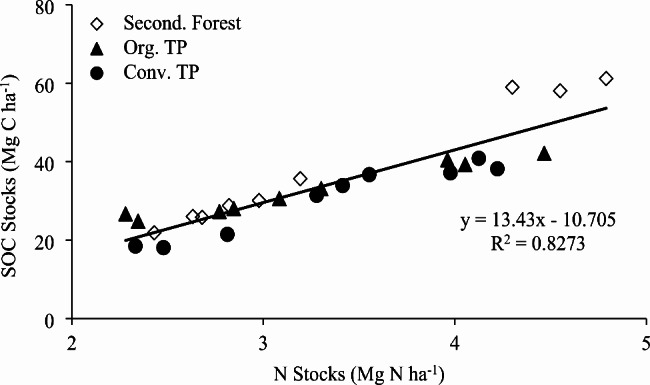
Relationship between SOC and N stocks accross soil layers and land-use types. ◇Second. Forest: Secondary Forest; ▲Org. TP: Organic Tea Plantation; ⬤Conv. TP: Conventional Tea Plantation

A positive correlation (R^2^ = 0.8273) was observed between N and SOC stocks across study sites, highlighting the strong connection between SOC sequestration and N accumulation (Fig. 3). Nevertheless, significantly higher levels of N and SOC stocks were observed in the 0–10 cm depth soil of the secondary forest, while the organic tea plantation and conventional tea plantation exhibited comparable capabilities in accumulating SOC and N.

## Discussion

### Soil properties following conversion of conventional tea plantation

The observed patterns of decreasing sand content and increasing clay content at all study sites suggest a gradual soil development, as evidenced by the cambic horizon (Bw) in the soil profiles (Table 1). These findings indicated that the soil texture had a limited influence on SOC and N stocks, as the study sites had similar soil textures owing to their proximity.

The conversion of the convnetional tea plantation to the secondary forest, as well as the varying management practices employed in tea plantations, have had significant effects on BD (Table 1). Notably, in the upper 10 cm layer, the secondary forest showed significantly lower BD compared to that of the conventional tea plantation. This observed low BD (0.37 ± 0.02 Mg m^− 3^) in the 0–10 cm layer of the secondary forest can be attributed to the accumulation of organic C resulting from a substantial amount of litterfall over the past 50 years, as suggested by Feng et al. (2019). In comparison, the secondary forest displayed significantly higher SOC content (160.95 ± 6.71 g kg^− 1^) than the conventional tea plantations (47.30 ± 4.95 g kg^− 1^), as shown in Table 2. Furthermore, Fig. 2a and b reveal a negative correlations between SOC and BD (R^2^ = 0.8238) and TN and BD (R^2^ = 0.8281), indicating that BD decreases as the SOC and TN contents increase. Grigal and Vance (2000) similarly proposed a general association between BD and SOM content. Ishizuke et al. (2021) provided further support for these findings, demonstrating that the conversion of cropland to forestland leads to a significant reduction in BD compared to nearby cropland. This change in land-use is believed to promote soil decompaction, aided by the activities of insects and invertebrates, as well as the expansion of root systems in the surface soils of forested areas (Ishizuke et al. 2021). Consequently, these results emphasize the positive impacts of reforestation in alleviating soil compaction and promoting overall soil health.

The high BD in the conventional tea plantation also indicated the impact of intensive agricultural practices on soil compaction. Low SOM and frequent cultivation activities after natural forests are converted to cultivated lands could result in increased BD in the surface soils (Abrishamkesh et al. 2011; Don et al. 2011; Kautsar et al. 2020; Murty et al. 2002; Wang et al. 2020). Cultivation practices, including decreased C input and increased C mineralization (Chen et al. 2016; Guan et al. 2015; Mancinelli et al. 2010; Sainju et al. 2008; Yimer et al. 2007), along with frequent fertilizing activities and multiple harvests per year, could have caused human-induced trampling, soil compaction, and structural degradation, resulting in higher BD in the tea plantations (Senapati et al. 2002). Igwe (2001) similarly found that compaction and structural degradation could lead to higher BD in the 0–20 cm layer of arable land.

The abandoned conventional tea plantation, upon succeeding to natural vegetation and converting to an organic tea plantation, exhibited varied soil pH levels (Table 1). The observed acidic pH in the surface soil of the secondary forest results from the release of hydrogen ions (H^+^) during the decomposition of organic matter (OM). Studies have indicated that the mineralization of OM results in the production of both organic and inorganic acids, thereby contributing to the overall soil acidity through the dissociation of H^+^ ions from acid functional groups (McCauley et al. 2009; Satrio et al. 2009).

Despite the significantly lower SOC content in the conventional tea plantation compared to the secondary forest, the soil pH in the 0–10 cm layer of the conventional tea plantation is remarkably low at 3.1 ± 0.1. This is likely attributed to the long-term application of soybean amendments and commercial nitrophosphate organic compound fertilizers. Applying soybean meal fertilizers could lead to soil acidification through the process of organic N nitrification and subsequent leaching of nitrate (NO_3_^−^) (Chang et al. 2008; Graham and Haynes 2005; Graham et al. 2002). Additionally, the long-term application of nitrogenous fertilizers such as ammonia sulfate, ammonium nitrate, ammonium sulfate nitrate, urea, calcium ammonium nitrate, and ammonium chloride in conventional tea plantations could further contribute to a decrease in soil pH owing to the nitrification of ammonium ions (Chin et al. 2010; Goh et al. 1987; Ma et al. 1990; Schmidt 1982).

### Changes in SOC and TN contents after converting to secondary forest

Converting a conventional tea plantation to a secondary forest has a noticeable effect on the SOC and TN contents, particularly in the 0–10 cm soil layer. In this layer, the secondary forest exhibits higher SOC and TN contents compared to the conventional tea plantation. This difference can be attributed to the increased C and N inputs derived from the higher litter production of the secondary forest. Feng et al. (2019) found that higher litterfall production leads to significant increases in SOC (200%), C (30%), and N (150%) contents in the topsoil (0–10 cm) following a 30-year period of forest restoration. Additionally, Yao et al. (2010) found a positive relationship between litterfall production and N levels in natural forests and mixed-tree plantations. Zhu et al. (2021) further highlighted the advantages of secondary forest conversion in the Nangou watershed in Ansai County, Shaanxi Province, China, by revealing higher SOC and TN contents in forests compared to croplands within the 0–10 cm soil layer. These findings emphasize the importance of secondary forest restoration to enhance SOM and nutrient stocks. In comparison with undisturbed forests, tea plantation cultivation leads to a reduction in C input owing to various factors such as harvesting, weeding, increased soil disturbance, and higher C mineralization (Chen et al. 2016; Guan et al. 2015; Mancinelli et al. 2010; Sainju et al. 2008; Yimer et al. 2007). These activities contribute to a decline in SOC and TN contents.

The significantly higher C/N ratio observed in the 0–10 cm layer of the secondary forest is a result of the long-term natural succession process following the conversion of tea plantations. This notable difference in the C/N ratio indicates a slower OM decomposition rate and a greater accumulation of SOC within the secondary forest. DeGryze et al. (2004) similarly demonstrated that native and successional forests generally exhibit higher C/N ratios compared to those of agricultural lands across different soil fractions. The secondary forest, characterized by an undisturbed environment and prolonged litter production, exhibits increased accumulation of SOC and N. The slower OM decomposition rate restricts soil microbial activities, leading to a greater retention of SOC. Zhu et al. (2017) and Xu et al. (2016) have emphasized the role of higher C/N ratios in reducing OM decomposition and facilitating SOC accumulation. Consequently, the higher C/N ratio observed in the topsoil of the secondary forest, in contrast to that in the conventional tea plantation, signifies a distinct soil ecosystem resulting from the natural succession process of the secondary forest. It implies a slower decomposition rate, enhanced storage of soil C, and the potential for long-term C sequestration within the secondary forest ecosystem.

### Changes in SOC and TN contents following conversion to organic farming

The management practices implemented in tea plantations can significantly influence the SOC and TN contents, particularly in the 0–10 cm layer. The subdtantial application of soybean amendments in the conventional tea plantation is considered to result in significantly higher SOC and TN contents compared to those in the organic tea plantation (Table 2).

Tea plants have a substantial demand for N. Therefore, the Agricultural Research and Extension Stations of Taiwan recommend adding 2,000–3,000 kg ha^− 1^yr^− 1^ of soybean meal fertilizer to enhance the N content in tea plantation soil. To fulfill this demand, tea farmers typically apply significant amounts of organic amendments rich in N supplements as part of their tea plantation management.

The C/N ratio is an important indicator of OM mineralization. When the C/N ratio falls within the range of 1–15, OM undergoes rapid mineralization, releasing N that becomes readily available for plant uptake (Brust 2019). Soybean meal fertilizer is commonly used as an N supplement in tea plantations in the Lugu area due to its lower C/N ratio (< 6) and faster mineralization compared to other organic fertilizers (He et al. 2019; Khan et al. 2019; Su et al. 2022; Van Kessel et al. 2000). According to He et al. (2019), soybean cake fertilizer contains 447.90 g kg^− 1^ of TC and 74.78 g kg^− 1^ of TN, having more than double the TC content and 3.5 times the TN content observed in animal manures. Therefore, applying soybean meal fertilizer not only contributes to the higher TN but also introduces a significant amount of C into the soil.

In addition to approximately 3,000 kg ha^− 1^ yr^− 1^ soybean amendments, the application of 4,500 kg ha^− 1^ yr^− 1^ nitrophosphate organic compound fertilizer as a nutrient supplement also serves as a source of SOC in the conventional tea plantation. In contrast, the organic tea plantation has only received approximately 4,400 kg ha^− 1^yr^− 1^ certificated organic fertilizer, a quantity similar to the application of nitrophosphate organic compound fertilizer in the conventinal tea plantation and seldom used soybean meal fertilizer. As a result, the conventional farming approach, which involves using similar intermediate practices, led to more significant increases in SOC and N content compared to the organic farming approach, primarily attributed to the application of more soybean amendments. Long-term organic farming is believed to sequester more soil C and N than conventional farming due to the accumulation of OM inputs and increased weed biomass (Kautsar et al. 2020). However, in this study, the significantly higher levels of SOC and TN in the 0–10 cm layer of the conventional tea plantation suggest that applying large amounts of soybean amendments in conventional tea plantations substantially impacts on SOC and N accumulation compared to organic farming practices.

Interestingly, in deeper soil layers, the organic tea plantation exhibits higher SOC and TN contents than the conventional tea plantation. The tea plantation age has been identified as a factor influencing SOC and TN contents and contributing to C and N accumulation (Chiti et al. 2018; Wang et al. 2016). The organic tea plantation, with a long cultivation history dating back from 1956 to 2014 before being converted to an organic system, could accumulate more SOC and TN in soil profile compared to those in the case of the conventional tea plantation. However, the significantly higher SOC and TN contents were only observed at the depth below 10 cm. Given the higher SOC and TN levels in the surface soil of the conventional tea plantation, it is assumed that the impact of applying soybean amendments in the conventional tea plantation has a stronger effect on SOC and TN contents than the age of the tea plantation in the 0–10 cm layer.

### Changes in SOC and N stocks after converting to secondary forest

Within a depth of 50 cm, the secondary forest (115.53 ± 7.23 Mg C ha^− 1^) showed significantly higher SOC stock compared to the conventional tea plantations (92.1 ± 8.54 Mg C ha^− 1^) (Table 3). The higher SOC stock in the secondary forest was primarily attributed to the markedly greater stock in the 0–10 cm layer, resulting from increased C and N inputs due to enhanced litter production in the upper soil layers. Moreover, less soil disturbance and lower SOC decomposition rate in forestlands can also contribute to greater SOC accumulation, thereby contributing to higher SOC stocks in the surface soil (Chen et al. 2016).

BD is a crucial parameter for calculating SOC and N stocks. A low BD can lead to reduced SOC and N stocks. Despite the secondary forest exhibiting significantly lower BD (0.37 ± 0.02 Mg m^− 3^) in the 0–10 cm layer compared to that in the tea plantations, its significantly higher SOC and TN contents still resulted in greater SOC and TN stocks after calculation (Table 3). In other words, even with its lower soil BD, the secondary forest exhibited 1.7 and 1.2 times higher SOC and TN stocks, respectively, compared to the conventional tea plantation. In the 10–30 cm layer, the conventional tea plantations benefited from a higher BD and exhibited greater SOC and N stocks in comparison to those of the secondary forest, even though the two land-use types had similar SOC and N contents in this layer (Table 2).

The findings demonstrate that reverting a conventional tea plantation into the secondary forest contributes to the recovery and reaccumulation of C. After 50 years of succession, the secondary forest successfully transformed, resembling a natural broadleaved vegetation state. This is a compelling example of the potential for restoring agricultural lands to their original natural vegetation or establishing afforested perennial vegetation as effective strategies for C reaccumulation. These results are consistent with those reported by DeGryze et al. (2004) and Post and Kwon (2000), which further support the feasibility and benefits of such approaches in increasing SOC stock.

While previous studies generally support the notion that secondary forests exhibit higher SOC and N stocks compared to those of tea plantations, other studies have observed the opposite trend, with tea plantations displaying higher SOC and N stocks than those of forests. Sohng et al. (2017) reported that tea plantations with higher BD had the highest SOC and N stocks compared to those of fern lands, pine plantations, and mature forests in Southwest Sri Lanka. Yulnafatmawita et al. (2020) found that tea plantations had higher SOC stocks than those of the nearby secondary forest in Mt. Talang, Solok Regency, West Sumatra, Indonesia. Chiti et al. (2018) also found that 31-year and 43-year tea plantations in Kenya exhibited increased SOC contents and stocks relative to those of primary and degraded forests.

### Changes in SOC and N stocks following conversion to organic tea plantation

Tea plantations with different management practices exhibited distinct distribution patterns of SOC and N stocks (Table 3). The variations in SOC and N stocks within the 50-cm layer between the two plantations were primarily influenced by the higher SOC and TN contents at a depth of 10 cm in the conventional tea plantation and below 10 cm in the organic tea plantation (Table 2).

As mentioned earlier, nine years of using soybean amendments in the conventional tea plantation increased SOC and TN levels in the 0–10 cm layer compared to the organic tea plantation. This discrepancy in fertilizer application likely contributed to the higher SOC and N stocks in the 0–10 cm layer of the conventional tea plantation (37.16 ± 3.49 Mg C ha^− 1^ and 3.70 ± 0.38 Mg N ha^− 1^, respectively) compared to the organic tea plantation (27.36 ± 3.08 Mg C ha^− 1^ and 2.57 ± 0.45 Mg N ha^− 1^, respectively). These results are consistent with previous studies that have shown the application of soybean meal fertilizer, rich in C and N, can effectively enhance C and N stocks (Gai et al. 2018; He et al. 2019; Khan et al. 2019; Shukla and Lal 2005). Gai et al. (2018) similarly observed that the long-term application of swine manure combined with chemical fertilizer increased SOC and N stocks in the 0–20 cm layer of the wheat-maize cropping system in the North China Plain. These results demonstrate that the application of soybean amendments in the convnetional tea plantation can effectively increase SOC and N stocks in the topsoil.

Organic farming systems is suggested to have higher SOC contents and stocks compared with nonorganic farming management systems (Gattinger et al. 2012). Nevertheless, the benefits of organic farming in increasing SOC and N stocks were not observed in the surface soil of the organic tea plantation. This might be attributed to the relatively brief eight-year period of implementing organic farming in the organic tea plantation, which may not have allowed for the sufficient accumulation of SOC and TN in the surface soil. Kautsar et al. (2020) found that C stocks in the surface layer increased significantly (by 15.5%) after 12 years of organic management compared to conventional fields. In contrast, no significant changes were observed after 4 or 8 years, indicating long-term organic rice farming increases soil C and N stocks and C and N mineralization in Japanese Andosols. Previous studies have indicated that the limited duration of organic farming does not significantly affect SOC and N stocks due to a common challenge during the transition to organic practices, where the soil microbial community adapts slowly to the increased nutrient cycling rates and N deficiency (Martini et al. 2004; Stringer et al. 2012). In contrast, the conventional tea plantation adopted practices resembling intermediate farming, which involved the application of soybean amendments, potentially enhancing the accumulation of TN and SOC in the 0–10 cm layer.

The age of the tea plantation had a positive impact on the SOC level, leading to higher SOC stocks in the 10–30 cm (40.63 ± 1.43 Mg C ha^− 1^) and 30–50 cm (29.48 ± 3.18 Mg C ha^− 1^) layers of the organic tea plantation compared to those of the conventional tea plantation. Chiti et al. (2018) similarly observed significantly higher SOC concentrations and stocks at various soil depths (0–5 cm, 15–30 cm, and 30–50 cm) in 31- and 43-year-old tea plantations compared to those in a 19-year-old tea plantation. Yulnafatmawita et al. (2020) suggested that cultivation aids in the sequestration of SOC, and SOC stocks tend to increase with the tea plantation age, even considering periodic tea leaf harvesting. Nevertheless, the impact of the tea plantation age on the increase in SOC and TN stocks was not evident in the 0–10 cm soil layer, suggesting that the application of soybean amenments in the conventional tea plantation has a more substantial influence on SOC and TN stocks within the 0–10 cm layer than the age of the tea plantation. Even though the age of the tea plantation didn’t seem to have a significant influence on N stocks in the deeper soil layers, it’s worth noting that the organic tea plantation still had higher N stocks compared to the conventional tea plantation.

### Relationships between SOC and N stocks

The significant and positive correlations observed between N and SOC stocks across all land-use types (Fig. 3) indicate that N stocks increase with SOC stocks (R^2^ = 0.8273). This finding is consistent with Deng et al. (2013), who also reported a positive correlation between SOC and N stocks during the conversion of farmlands to grasslands in Shaanxi Province, China. Furthermore, the higher levels of N and SOC stocks observed in the 0–10 cm depth soil demonstrate that the secondary forest has a higher potential to sequestrate more SOC and N in the surface soil compared to the conventional tea plantation. This is supported by the high accumulation of SOC and N and the greater C/N ratio in the surface soil of the secondary forest (Tables 2 and 3).

In contrast, unlike the significant increase in SOC and N stocks observed in the 0–10 cm layer when converting a conventional tea plantation to a secondary forest, the conventional tea plantation transitioning to an organic tea plantation exhibited a comparable capability to sequestrate SOC and N at all soil depths. Although variations existed in the contributions of N and SOC stocks between the surface soil and subsoil in both types of tea plantations, these differences were not statistically significant after accounting for their respective effects.

## Conclusions

Within a 50-cm depth, the secondary forest exhibited significantly higher SOC stock compared to that of the conventional tea plantation. However, no significant differences in N stocks were seen between the two study sites. The notable disparity in SOC and N stocks between the secondary forest and conventional tea plantation was primarily observed in the 0–10 cm layer and was attributed to the C and N inputs derived from high litter production over a 50-year succession period in the secondary forest. In contrast, the higher BD in the conventional tea plantation resulted in higher SOC and N stocks than those in the secondary forest in the 10–30 cm layer.

Two tea plantations displayed contrasting patterns in the distribution of soil organic SOC and N stocks. The higher levels of SOC and N contents and stocks were observed at a depth of 10 cm in the conventional tea plantation and below 10 cm in the organic tea plantation. The higher stocks in the conventional tea plantation were attributed to the use of soybean meal fertilizer and soybean flour, while the brief period of implementing organic farming in the organic tea plantation did not lead to the substantial accumulation of SOC and TN in the surface layer. In contrast, the conventional tea plantation, employing similar intermediate farming practices, increased SOC and N contents and stocks in the surface soil compared to the organic tea plantation.

Furthermore, the age of the tea plantation positively impacted SOC levels, resulting in higher SOC stocks in deeper soil layers for the organic tea plantation. These findings highlight the significance of fertilizer management and tea plantation age in determining SOC and N stocks, emphasizing the importance of sustainable practices in tea cultivation. However, despite these differences, there were no significant disparities in SOC and N stocks between the two plantations within the 50 cm depth. These findings suggest that the conversion to organic practices did not lead to notable increases in SOC and N stocks in Nantou County, Taiwan.

### Electronic supplementary material

Below is the link to the electronic supplementary material.

Supplementary Material 1

## Data Availability

The datasets used and analyzed during the current study are available from the corresponding author (CPC, chioupinchen@ntu.edu.tw) on reasonable request.
