# Supplementary material for: Changes in soil organic carbon and nitrogen stocks in organic farming practice and abandoned tea plantation
Source: Bot Stud. 2023 Sep 28;64:28. doi: 10.1186/s40529-023-00401-z (PMC10533459; doi:10.1186/s40529-023-00401-z)
Supplement: Supplementary file 1 — Supplementary Material 1 [file 40529_2023_401_MOESM1_ESM.pdf]

SCRIBENDI

Scribendi Inc.
304-405 Riverview Drive
Chatham, Ontario N7M 0N3 Canada

Receipt

Customer

Chiou-Pin Chen
No.12, Sec. 1, Qianshan Rd.,
Zhushan Township
Nantou County
Nantou County
557004

Date: 2023-07-25
Task No.: 960005
Returned

Words	Description	TOTAL
7,473	Academic Editing in 2 days<br>Submitted files:<br>Botanical studies - 2nd revised 20230725 for....docx	USD 291.70

	Sub Total	USD 291.70
	Shipping	USD 0.00
Tax Rate(s)	None	USD 0.00
	TOTAL	USD 291.70

Payments

Methods: Stripe
Amount: US$ 291.70
Date: Tue, 25 Jul 2023 16:27:06 GMT

Office use only

PAID IN FULL

The client is responsible for remitting all local taxes and fees to the relevant authorities.

Thank you for your business!

All orders subject to our terms of service: https://www.scribendi.com/terms

GST/HST #: 89219 5702 RT0001

QST #: 1225989199 TQ0001
